# Supplementary material for: SyNDI: synchronous network data integration framework
Source: BMC Bioinformatics. 2018 Nov 6;19:403. doi: 10.1186/s12859-018-2426-5 (PMC6219086; doi:10.1186/s12859-018-2426-5)
Supplement: Supplementary file 5 — Files of the example presented in Additional file 4. This additional file is a zip package that contains all files used in Additional file 4. Detailed descriptions of each of these files are presented in a README file included in this zip package. (GZ 30411 kb) [file 12859_2018_2426_MOESM5_ESM.gz › SyNDITools/Meme2Fimo/meme_4.9.0_4.tar.gz!/meme_4.9.0/doc/bfile-format.html]

Meta-MEME - Background model format


# Background model format

The format for n-order Markov background models is as follows.

The file must contain one line for each combination of
1, 2, ..., n-1 letters in the alphabet. The DNA alphabet is **ACGT**
and protein the protein alphabet is **ACDEFGHIKLMNPQRSTVWY**.

Each line must contain the letter combination followed by the
letter combination's frequency (probability).
All other lines in the file are ignored, including
comment lines starting with '#'.

For example, a 0-order Markov model <file> might contain:

```
# tuple   frequency_non_coding
a       0.324
c       0.176
g       0.176
t       0.324
```

A 1-order Markov model <file> might contain:

```
# tuple   frequency_non_coding
a       0.324
c       0.176
g       0.176
t       0.324
# tuple   frequency_non_coding
aa      0.119
ac      0.052
ag      0.056
at      0.097
ca      0.058
cc      0.033
cg      0.028
ct      0.056
ga      0.056
gc      0.035
gg      0.033
gt      0.052
ta      0.091
tc      0.056
tg      0.058
tt      0.119
```

**NOTE:**
You can create a background model file from any
FASTA sequence file using the
`fasta-get-markov` command.
